# Insight into the charge transfer in particulate Ta_3_N_5_ photoanode with high photoelectrochemical performance†

**DOI:** 10.1039/c6sc00245e

**Published:** 2016-03-16

**Authors:** Zhiliang Wang, Yu Qi, Chunmei Ding, Dayong Fan, Guiji Liu, Yongle Zhao, Can Li

**Affiliations:** a State Key Laboratory of Catalysis, Dalian Institute of Chemical Physics, Chinese Academy of Sciences, Dalian National Laboratory for Clean Energy, The Collaborative Innovation Center of Chemistry for Energy Materials (iChEM) Zhongshan Road 457 Dalian 116023 China canli@dicp.ac.cn; b University of the Chinese Academy of Sciences Beijing 100049 China

## Abstract

Charge separation is one of the most critical factors for generating solar fuels *via* photoelectrochemical water splitting, but it is still not well understood. This work reveals the fundamental role of charge transfer in photoanodes for achieving high charge separation efficiency. Specifically, we fabricated a particulate Ta_3_N_5_ photoanode by a bottom-up method. By improving the charge separation with refined necking treatment, the photocurrent is increased by two orders of magnitude. The charge separation efficiency (*η*_sep_) is analyzed by dividing it into charge generation efficiency (*Φ*_gene_) and transportation efficiency (*Φ*_trans_). Necking treatment is found to substantially improve the electron transfer. Transient photovoltage (TPV) measurements based on the Dember effect is used to confirm the benefit of necking treatment in improving the charge transportation. The superior electron transfer in the necked-Ta_3_N_5_ electrode is further evidenced by the facile electron exchange reaction with the ferri/ferrocyanide redox couple. Moreover, cobalt phosphate is found to promote both charge separation and surface reaction, resulting in a photocurrent of 6.1 mA cm^−2^ at 1.23 V *vs.* RHE, which is the highest response for a particulate photoanode.

## Abstract 2

Charge transfer has been demonstrated to have a fundamental role in particulate Ta_3_N_5_ electrode for achieving high efficient photoelectrochemical water oxidation.

## Graphical abstract

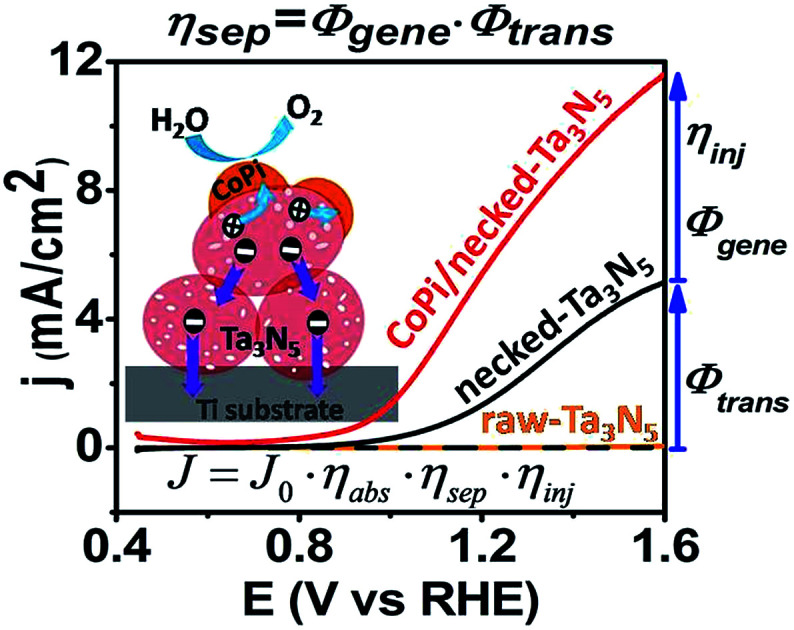

## Introduction

Photo-induced water splitting paves a promising way for the production of renewable solar fuels by converting solar energy to hydrogen directly. To achieve this conversion process, photocatalysis (PC) and photoelectrocatalysis (PEC) are viable technological choices.^1–4^ Many kinds of materials have shown photoresponse in PC but failed in PEC due to the issue of electrode fabrication.^2,5,6^

In PC, an efficient charge separation in particles can usually lead to fast reaction at the semiconductor/liquid interface with the assistance of a suitable cocatalyst.^7^ In PEC, however, photogenerated electrons must transfer through the film and be collected by the substrate to match the surface reaction. Thus, the charge transportation in the films also plays a fundamental role in the whole PEC process,^8^ and the interfaces of particle–particle (PP) and particle–substrate (PS) are critically important for charge transportation. To ensure good connections at these interfaces, semiconductor is usually epitaxially grown on a conductive layer *in situ* by, for instance, hydrothermal,^9^ chemical bath,^10^ and vapour deposition methods.^11,12^ Electrodes of low-dimensional structure^13,14^ or host–guest structure^15,16^ are intentionally designed to improve the charge transportation efficiency in the electrode so as to decrease the electron–hole recombination. However, most previous researches focus on charge transfer in the semiconductor particles since it determines the generation of separated electron–hole pairs,^17–19^ and little attention has been paid on clarifying the charge transfer process in the film, which also plays a determinable role during the photoelectrochemical reaction.

Transient photovoltage (TPV) can provide direct insight into the charge transfer process through the electrode. Upon illumination, Dember photovoltage is generated^20^ which stems from the diffusion difference of photogenerated electrons and holes. Typically, the gradient distributed light in the film excites photogenerated electron–hole pairs in gradient concentration^21,22^ which will cause them to diffuse from a high concentration region (surface, high light intensity) to the low concentration region (bulk, low light intensity) at different velocities. Then the electron–hole pairs drift apart and electric field builds up; the decay process of the photovoltage can reveal the charge transfer process in the film.

Cyclic voltammetry (CV) in the dark can also reveal the electron transfer in electrodes.^23^ For the cathodic reaction, electrons must transfer through the films before exchanging with an efficient redox couple, *e.g.* Fe(CN)_6_^3−^/Fe(CN)_6_^4−^. The magnitude of the cathodic current and potential of the reduction peak can reveal the electron transportation in the film. In addition, a particulate electrode with well-connected film can result in porous structure which provides a large electrochemical surface area for reaction. This area is proportional to the capacitance of the Helmholtz layer which can be determined from CV^24^ and so we can evaluate the charge transportation in the film by an electrochemical method.

Suitable materials and fabrication methods of the electrodes are important for us to pinpoint the issue of charge transportation. In terms of materials, semiconductors with long free paths are the best candidates because it permits us to focus on the charge transfer at the PP interfaces regardless of the charge diffusion in the semiconductor crystals. Ta_3_N_5_ is reported to have a diffusion length of ∼10^3^ nm,^25^ indicating a long life time of photogenerated charges. Moreover, Ta_3_N_5_ has demonstrated an outstanding photocatalytic oxygen evolution activity,^26^ indicating an effective charge separation in Ta_3_N_5_ crystals. Also, the excellent light harvesting ability of Ta_3_N_5_ makes it an appealing material for photoelectrochemical water splitting with a potential solar energy conversion efficiency of 15% under AM 1.5 G sunlight.^27^ For the fabrication of the electrode, a bottom-up method, that is by depositing as-prepared semiconductor particles on the conductive substrate to form a particulate electrode, can make it more facial so as to regulate the charge transfer at the PP and PS interfaces without influencing the intrinsic properties of the semiconductor such as light absorption, carrier concentration *etc.* Electrophoretic deposition (EPD) method is an alternative choice. A wide range of semiconductors can be fabricated into electrodes with controllable thickness by EPD, such as Fe_2_O_3_,^28^ BiVO_4_,^29^ Ta_3_N_5_,^30^ TaON,^31,32^ while post-necking treatment has been found to be powerful in improving the PEC response.^30–34^ Some explanation to the possible function of necking treatment has been proposed,^31^ but more stringent evidences are required to identify the effects of this modification.

Herein, we take Ta_3_N_5_ as a demonstration to show the influence of charge transportation on the PEC performance of a particulate Ta_3_N_5_ electrode. By optimizing the substrate, precursor concentration, temperature of necking treatment and cocatalyst loading, we have achieved the highest photocurrent for electrodes fabricated by EPD. The TPV based on Dember photovoltage and CV measurements are used to clarify the critical effect of necking treatment on charge transfer at the interfaces of particle–particle and particle–substrate.

## Experimental

### Ta_3_N_5_ powder synthesis

Ta_2_O_5_ powder (Amresco Chemical, ≥99.99%) was immersed in water, then it was dried and annealed in air at 800 °C for 2 h, prior to being nitrided in ammonia flow (250 sccm) at 950 °C for 15 h.

### Ta_3_N_5_ electrode fabrication

The as-synthesized Ta_3_N_5_ powder (50 mg) was dispersed into 50 mL acetone (Kemeol, ≥99.5%) under ultrasonic treatment for 10 min. Then 20 mg iodine was dissolved into the suspension to make it suitable for EPD. Ti foil (1 cm × 2 cm) was used as substrate after washing in 1 M HF aqueous, pure water and anhydrous ethanol. A piece of fluorine-doped tin oxide (FTO, Nippon Glass Sheet) glass (2 cm × 3 cm) is used as the counter electrode with the conductive layer facing towards the Ti foil at a distance of 1 cm. The Ta_3_N_5_ crystals were deposited to the Ti foil at a bias of 20 V for 1 min.

### Necking treatment

20 mM TaCl_5_ (Alfa Aesar, ≥99.99%) was dissolved into anhydrous methanol (Sinopharm Chemical Reagent, ≥99.5%) and 10 μL TaCl_5_ solution was then dropped onto the raw Ta_3_N_5_ electrode (denoted as raw-Ta_3_N_5_, area of 1–1.3 cm^2^) five times (50 μL in total) to form the TaCl_5_ treated Ta_3_N_5_ electrode (denoted as TaCl_5_–Ta_3_N_5_ electrode) based on ref. 40. Then the TaCl_5_–Ta_3_N_5_ electrode was calcined at 600 °C (5 °C min^−1^) for 60 min under NH_3_ flow (100 sccm), and the as-prepared electrode is donated as necked-Ta_3_N_5_. For comparison, the raw-Ta_3_N_5_ electrode was also heated in the same way without TaCl_5_ solution (denoted as heated-Ta_3_N_5_).

### Cobalt phosphate deposition

5 mM Co(NO_3_)_2_ (Sinopharm Chemical Reagent, ≥99.0%) was dissolved into 0.5 M NaPi buffer solution (pH 6.8). Then CoPi was electrochemically deposited onto the Ta_3_N_5_ electrode under simulated light (AM 1.5 G, 100 mW cm^−2^) illumination at a current density of 10 μA cm^−2^.

### Characterization

The absorption spectra from 350 to 800 nm were taken on Cary 5000 UV-VIS-NIR spectrophotometer (JASCAO) equipped with an integrated sphere. X-Ray diffraction (XRD) patterns were recorded on Rigaku D/Max-2500/PC powder diffractometer operating at 40 kV and 200 mA with Cu-Kα radiation (*λ* = 0.154 nm) at a scanning rate of 5° min^−1^. The morphology of the electrodes was imaged by a Quanta 200 FEG scanning electron microscope (SEM). High-resolution transmission electron microscopy (HRTEM) images were obtained on Tecnai G2 F30 S-Twin (FEI Company) with an accelerating voltage of 300 kV. X-Ray photoelectron spectroscopy (XPS) was recorded on VG ESCALAB MK2 spectrometer with monochromatic Al-Kα radiation (12.0 kV, 240 W). All the bonding energies were corrected with reference to the C 1s (284.8 eV) signal.

### Transient photovoltage measurement

The transient photovoltage (TPV) was measured with a pulsed laser (355 nm, 5 ns) using a Ta_3_N_5_ device (see ESI for details†). The average power was 122 mW unless otherwise stated. The signals were read from an oscilloscope (Tektronix TDS 3012C).

### (Photo)electrochemistry measurement

Cyclic voltammetry was measured without illumination in an electrolyte of NaOH (1 M) or NaOH (1 M)–K_3_Fe(CN)_6_ (0.25 M) aqueous solution at a scan rate of 100 mV s^−1^ in the range of −1.4 to 0.4 V *vs.* SCE. For the electrochemical area measurement, the scan rate was varied from 20 to 500 mV s^−1^ in the range of −0.1 to 0.2 V *vs.* SCE in 1 M NaOH aqueous solution.

The photocurrent was recorded under simulated light (AM 1.5 G, 100 mW cm^−2^) at a scan rate of 50 mV s^−1^ from −0.8 V to 0.6 V *vs.* SCE in 1 M NaOH aqueous solution (pH 13.6).

The incident photon-to-current conversion efficiency (IPCE) was measured under monochromatic light irradiation provided by a tungsten lamp equipped with a monochromator (CROWNTECH, QEM24-D 1/4 m Double).

Faradaic efficiency was tested by recording the photocurrent and the generated O_2_ simultaneously. The O_2_ evolution was evaluated by gas chromatography (GC, Agilent 7890a GC) with a 5 Å molecular sieve column. Argon carrier gas with a velocity of 10.0 mL min^−1^ was used to purge the working electrode compartment to carry the evolved gases to GC for analysis. The quantity and retention time of the gases were calibrated with a series of standard gas samples.

All the (photo)electrochemical tests were conducted on an Ivium potentiostat/galvanostat in a three-electrode system with a quartz window. A piece of platinum foil (2 cm × 2 cm) was used as counter electrode and saturated calomel electrode (SCE, 0.241 V *vs.* RHE) used as the reference electrode. The potential was converted to the reversible hydrogen electrode (RHE) scale by the Nernst equation as below:

1 *E*(RHE) = *E*(SCE) + 0.059pH + 0.241

## Results and discussion

**Fig. 1:**
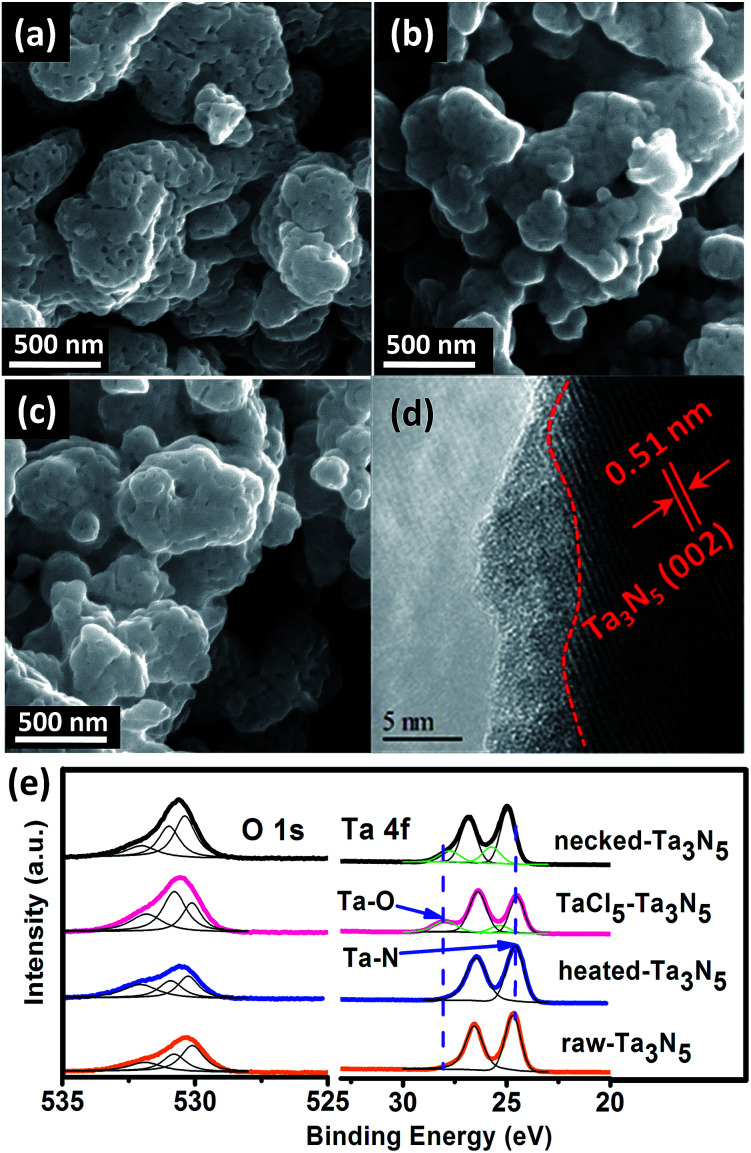
SEM images of (a) raw-Ta_3_N_5_ electrode, (b) TaCl_5_–Ta_3_N_5_ electrode, and (c) necked-Ta_3_N_5_ electrode. (d) HRTEM image of necked-Ta_3_N_5_ electrode. (e) XPS spectra of O 1s and Ta 4f for raw-Ta_3_N_5_ (orange), heated-Ta_3_N_5_ (blue), TaCl_5_–Ta_3_N_5_ (pink) and necked-Ta_3_N_5_ (black) electrodes.

The morphology of the electrodes was first characterized by SEM. The SEM image of a cross-section view shows that the as-prepared electrode has a thickness of 5–7 μm (Fig. S1†). Top-view images show that the surface of porous raw Ta_3_N_5_ (Fig. 1a) is covered with an amorphous layer after treating with TaCl_5_ solution (Fig. 1b). The following heat treatment seems to retrieve the smooth surface of Ta_3_N_5_ but boundaries between particles become less obvious compared to those on raw-Ta_3_N_5_ electrode (Fig. 1c). The HRTEM image confirms the existence of a 1–5 nm amorphous layer (Fig. 1d). This amorphous tantalum species may bridge the adjacent Ta_3_N_5_ particles. The XRD patterns (Fig. S2†) show no change of the Ta_3_N_5_ crystals with necking treatment, however, the surface change was revealed in XPS spectra (Fig. 1e). There is no prominent difference between raw-Ta_3_N_5_ and heated-Ta_3_N_5_ electrodes but for the TaCl_5_–Ta_3_N_5_ electrode, another peak at 28.1 eV was observed, in agreement with the reported Ta 4f_5/2_ signal in tantalum oxide.^35,36^ It is inferred that the amorphous layer in Fig. 1b may be tantalum oxide. Since it was covered on the raw-Ta_3_N_5_ electrode, the intensity of O 1s (530.7 eV) became stronger, and the intensity of Ta 4f_7/2_ (24.6 eV) became weaker after treating with TaCl_5_ solution, indicating the screening effect of the amorphous tantalum oxide. It is also inferred that Ta 4f_7/2_ at 24.6 eV arises from Ta–N bonds. After necking treatment, the intensity of O 1s (530.7 eV) ascribed to surface absorbed oxygen species is prominently decreased compared to the TaCl_5_–Ta_3_N_5_ electrode, with Ta 4f_5/2_ ascribed to Ta–O shifted from 28.0 to 27.6 eV, and Ta 4f_7/2_ ascribed to Ta–N bond shifted from 24.6 to 25.0 eV for the necked-Ta_3_N_5_ electrode. It is suggested that a nitrogen-doped tantalum layer has been formed. Because the Ta–N bond is more covalent than the Ta–O bond, nitrogen doping of tantalum oxide during necking treatment will shift the binding energy of Ta 4f to lower energy.^36–38^

**Fig. 2:**
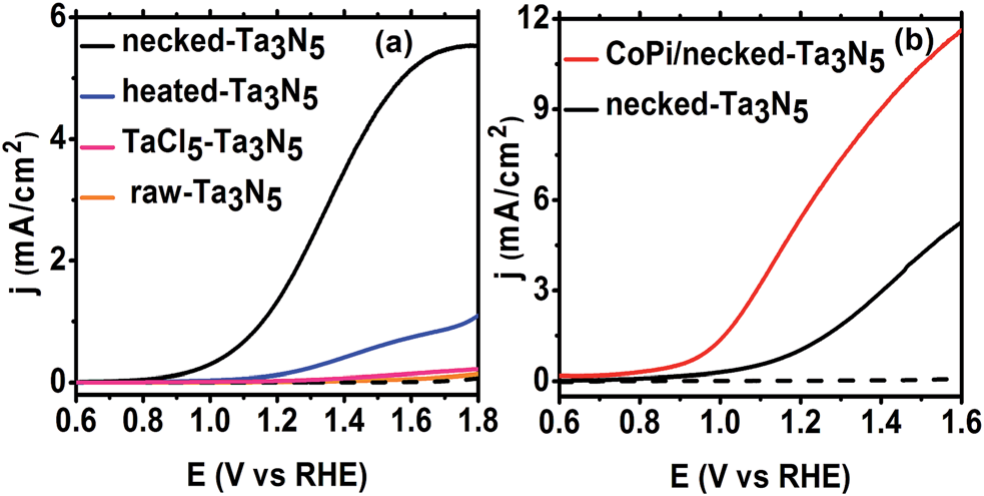
Current–potential (*j*–*E*) curves of (a) raw-Ta_3_N_5_ (orange), TaCl_5_–Ta_3_N_5_ (pink), heated-Ta_3_N_5_ (blue), necked-Ta_3_N_5_ (black) and (b) CoPi/necked-Ta_3_N_5_ (red) electrode. Electrolyte: 1 M NaOH aqueous solution. Illumination: 100 mW cm^−2^, AM 1.5 G.

**Fig. 3:**
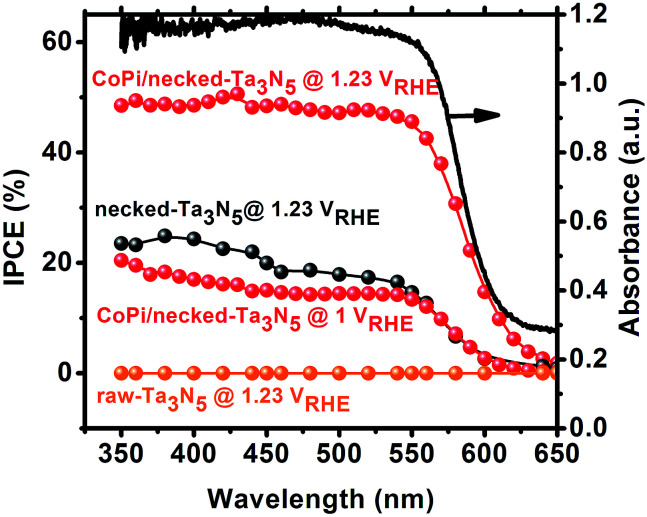
IPCE of raw-Ta_3_N_5_ (orange), necked-Ta_3_N_5_ (black) and CoPi/necked-Ta_3_N_5_ (red) electrodes. The absorption spectrum (black solid curve) for raw Ta_3_N_5_ is presented for comparison.

Despite the small changes of the morphology of Ta_3_N_5_ particles, the PEC performance shows a substantial difference with necking treatment (Fig. 2a). The raw-Ta_3_N_5_ electrode exhibits notoriously low photoresponse, and so does the TaCl_5_ treated electrode. However, after necking treatment, the photocurrent of the necked-Ta_3_N_5_ electrode increases to 1.56 mA cm^−2^ from 9 μA cm^−2^. For the necking treatment, the TaCl_5_ concentration and post-heating temperature have great influence on the photoresponse of the necked-Ta_3_N_5_ electrode (Fig. S3†). The optimized TaCl_5_ concentration is 15–20 mM (concentration at 20 mM gives the best repeatability) and the best post-heating temperature is 600 °C for the refined necking treatment. At high temperature, the particles will have strong connection with each other at the PP and PS interfaces. However, NH_3_ has strong capability of reduction at high temperature, and can damage the normally used FTO substrate.^28,31^ Some metal based candidates were chosen and Ti foil gave the best result (Fig. S4†). The benefit of calcination is also confirmed by the improved photoresponse of heated-Ta_3_N_5_ compared to the raw-Ta_3_N_5_ electrode. Further cocatalyst loading, *e.g.* CoPi,^39^ on necked-Ta_3_N_5_ electrode efficiently accelerates the water oxidation to an optimized current of 6.1 mA cm^−2^ at 1.23 V *vs.* RHE (Fig. 2b), which is higher than most reported results fabricated by the EPD method.^30,31,40,41^ In Fig. 3, IPCE action spectra show the quantum efficiency at different irradiation wavelengths, which are in agreement with the absorption of Ta_3_N_5_. The raw-Ta_3_N_5_ electrode shows very low IPCE, but after the refined necking treatment, it dramatically increases to more than 20%. Also, the IPCE is much higher by loading CoPi or increasing the bias to accelerate the consumption of photogenerated charges.

The refined necking treatment plays a key role in boosting the photoanodic performance of the particulate Ta_3_N_5_ electrode and it is also essential for the cocatalyst functioning Ta_3_N_5_ photoanode. Thus we endeavour to pinpoint the exact role of necking treatment by analysing the reaction at the semiconductor/liquid interface and charge transfer at PP and PS interfaces on the particulate Ta_3_N_5_ electrode.

**Fig. 4:**
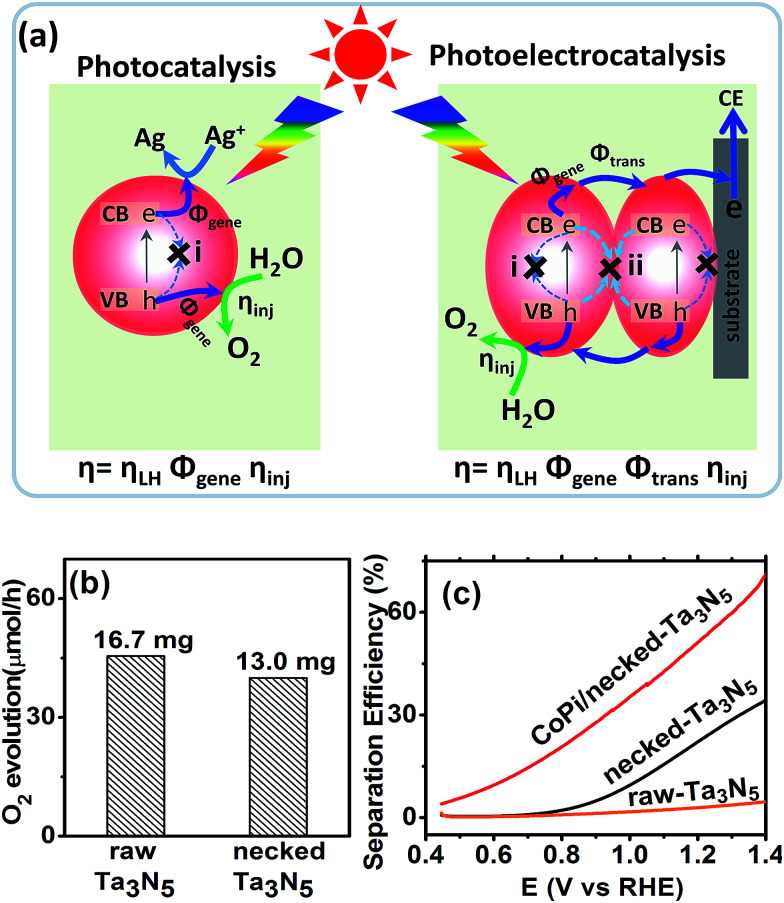
(a) Schematic comparison of O_2_ evolution processes *via* photocatalysis (AgNO_3_ as electron scavenger) and photoelectrocatalysis. (b) Photocatalytic activity of the Ta_3_N_5_ powder peeled from raw-Ta_3_N_5_ and necked-Ta_3_N_5_ electrodes. The mass of the photocatalyst is listed above the column, respectively. (c) Charge separation efficiency of raw-Ta_3_N_5_ (orange), necked-Ta_3_N_5_ (black) and CoPi/necked-Ta_3_N_5_ electrodes (red).

For an electrode made from powder, necking treatment may have an influence on both the intrinsic property of Ta_3_N_5_ particles and the connection among particles. In order to elucidate the possible change of Ta_3_N_5_ crystals, the absorption spectra and photocatalytic activity (see ESI for details†) were measured. The absorption spectrum of Ta_3_N_5_ shows little change after necking treatment (Fig. S5a†), indicating that the necking treatment does not influence the intrinsic light harvest in Ta_3_N_5_. From the absorption spectra, the marginal photocurrent of the electrode was evaluated to be ∼12.6 mA cm^−2^ under simulated sunlight (AM 1.5 G, 1 sun) (Fig. S5†). The influence of necking treatment to the Ta_3_N_5_ particles were further ascertained by photocatalytic O_2_ evolution. In a PC reaction (Fig. 4a), light is harvested in the Ta_3_N_5_ crystal and photogenerated charges drift to the active sites to take part in the surface reaction. The proportion of photogenerated charges that reach the surface reaction sites to those generated in Ta_3_N_5_ particles upon illumination is referred as charge generation efficiency (*Φ*_gene_), which is basically the charge separation efficiency in the Ta_3_N_5_ crystal. To preventing possible confusion with the separation efficiency in the electrode (as will be mentioned below), we define it as generation efficiency for the crystal. This generation efficiency is largely determined by the bulk property of the crystal. The proportion of photogenerated holes that take part in the (electro)chemical reaction to those arriving at the reaction sites is referred as injection efficiency (*η*_inj_), which is influenced by the surface property of Ta_3_N_5_. When the surface is not preferred for catalytic water oxidation, although sufficient holes reach the reaction sites, they cannot be consumed in time and *η*_inj_ will be less than unity. In the presence of efficient electron scavengers, the influence of electrons in the crystal is limited and the PC reaction can be used to evaluate the intrinsic properties of the Ta_3_N_5_ particles, such as the charge generation in the crystals and hole injection on the surfaces.^42^ The PC activities of the Ta_3_N_5_ powder from the raw-Ta_3_N_5_ electrode and necked-Ta_3_N_5_ electrode were evaluated with AgNO_3_ as sacrificial reagent.^26^ As is shown in Fig. 4b, the amount of released O_2_ for necked-Ta_3_N_5_ has slightly decreased, implying that necking treatment has limited influence on the intrinsic property of Ta_3_N_5_.

Then we focussed on the impact of necking treatment to the electron transfer at the PP and PS interfaces in the Ta_3_N_5_ electrode. In a PEC reaction (Fig. 4a), electrons and holes should be considered simultaneously since the electrons need to transfer through the Ta_3_N_5_ films during the PEC water oxidation. The transportation of photogenerated charges will be another factor that limits the PEC performance.

As it is reported, the final photocurrent is determined by the following expression:^43,44^

2 *J* = *J*_0_*η*_LH_*η*_sep_*η*_inj_

where *J*_0_ is the theoretical photocurrent, *η*_LH_ is the light harvest efficiency, *η*_inj_ is the charge injection efficiency as defined above, and *η*_sep_ is separation efficiency in the electrode as defined in ref. 43.

The absorption and PC water oxidation measurement have revealed that necking treatment has little influence on light harvest (*η*_LH_) and surface reaction (*η*_inj_). Hence, the difference of charge separation (*η*_sep_) of the electrode should be responsible for the huge difference of the PEC activity. Taking H_2_O_2_ as hole scavenger,^43^ the charge separation efficiency is calculated. From Fig. 4c, it is revealed that the charge separation in raw-Ta_3_N_5_ electrode is inefficient, but necking treatment improves *η*_sep_ to more than 30% at the bias of 1.3 V *vs.* RHE. Interestingly, we found that CoPi/necked-Ta_3_N_5_ electrode has an even higher *η*_sep_ of 60% at 1.3 V *vs.* RHE.

A higher separation efficiency means less electron–hole recombination in the electrode. For a photoelectrocatalytic reaction occurring on a particulate electrode (Fig. 4a), the recombination may occur *via* two routes: (i) recombination in the particles through, for example, bulk defects or surface states; (ii) recombination at the interfaces of particles. Thus the measured separation efficiency should be dependent on the charge generation in particles and transfer among particles under illumination:

3 *η*_sep_ = *Φ*_gene_*Φ*_trans_

where *Φ*_gene_ is the charge generation efficiency as mentioned in Ta_3_N_5_ crystal, relating to recombination process (i), and *Φ*_trans_ is the transportation efficiency for photogenerated charges transferring through PP and PS interfaces before arriving at the conductive substrate from the origin,^45^ relating to recombination process (ii).

CoPi has been reported to suppress the surface states, and more long-lived photogenerated holes can survive in the particles.^46^ Thus, the *Φ*_gene_ is increased and this leads to the cocatalyst promoted charge separation for the CoPi/necked-Ta_3_N_5_ electrode (Fig. 4c). For the necked-Ta_3_N_5_ electrode, the previous absorption (Fig. S5†) and PC activity (Fig. 4b) indicates a similar *Φ*_gene_. Thus, the dramatically improved *η*_sep_ for necked-Ta_3_N_5_ electrode should stem from the improved *Φ*_trans_ upon necking treatment.

**Fig. 5:**
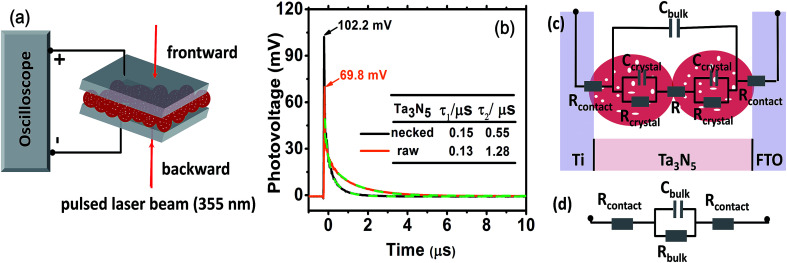
(a) Schematic setup for TPV test. (b) TPV spectra of raw-Ta_3_N_5_ (orange) and necked-Ta_3_N_5_ electrode (black) with dual exponential fitted curves (green dashed curves). The inset table shows the lifetimes of the two electrodes, respectively. (c) Equivalent circuit of the device. (d) The simplified circuit of (c). *R*_contact_ is the serial resistance at the particle–substrate interfaces; *R*_bulk_ is the total resistance in Ta_3_N_5_ films including the resistance in Ta_3_N_5_ particles (*R*_crystal_) and at particle–particle interfaces (*R*); *C*_bulk_ is the capacity of the parallel-plate capacitor built by Ti and FTO.

To consolidate the conclusion, prototypical devices fabricated from Ta_3_N_5_ electrodes (Fig. 5a, see ESI for details†) were used to delve into the charge transportation in the electrode by TPV. In Fig. 5b, the necked-Ta_3_N_5_ based device shows higher transient photovoltage and faster decay process than the raw-Ta_3_N_5_ based device. A linear response of current-bias (Fig. S6†) confirms the ohmic contact at the interfaces of PS.^20^ Thus, the detected photovoltage plausibly stems from the Dember effect and this was further confirmed by the relationship between the direction of the induced laser and electric field. For the Dember effect, holes always stay closer to the top layer, and the direction of the electric field is always consistent with the direction of the laser beam (Fig. S7a†), and the intensity of the Dember voltage is determined by the amount of separated charges. Light of stronger intensity will induce more separated charges and hence a higher photovoltage (Fig. S7b†). When the intensity of the induced light is the same, and the same amount of charges are generated, a higher photovoltage indicates better charge separation. Thus the higher photovoltage of the necked-Ta_3_N_5_ device indicates that it shows better charge separation which is in accordance with the result in Fig. 4.

When light is removed, the electron–hole pairs will recombine, leading to a dynamic decay of the Dember photovoltage at the time scale of μs (Fig. 5b, solid line). Two plausible recombination processes mentioned above are involved in the decay process. Dual exponential curves (Fig. 5b, dashed line) are fitted with a fast decay process (recombination in Ta_3_N_5_ crystals, lifetime of *τ*_1_) and a slow one (recombination at interfaces, lifetime of *τ*_2_). The two electrodes have approximately the same *τ*_1_ values, confirming again that the Ta_3_N_5_ particles on both electrodes have similar charge generation efficiency (*Φ*_gene_) as the PC result reveals in Fig. 4a. However, the necked-Ta_3_N_5_ electrode has lower *τ*_2_, indicating that the electron–hole pairs more readily recombine through the interfaces in the TPV devices.

In order to clarify the result of TPV further, an equivalent circuit (EC) based on the transmission line model^47^ (Fig. 5c) was used to simulate the decay process in the device. Because there is no semiconductor/electrolyte interface, only three processes are considered: (i) the electron transport resistance in the Ta_3_N_5_ crystals (*R*_crystal_), (ii) the electron transport resistance among particles (*R*) and particles–substrate (*R*_contact_), (iii) a capacitive charging to the porous Ta_3_N_5_ matrix (*C*_bulk_). For the Ta_3_N_5_ crystal, the conduction band or the defect energy level can accommodate electrons which can behave like a capacitor (*C*_crystal_). *C*_bulk_ can be estimated to be at the order of 10^−5^ F cm^−2^, while *C*_crystal_ is around 10^−8^ F cm^−2^ (see ESI for details†). Thus, *C*_crystal_ is much smaller compared to *C*_bulk_ and the series of *C*_crystal_ make it even smaller, so that we can ignore *C*_crystal_. So the equivalent circuit in Fig. 5c can be reduced to that in Fig. 5d, including the resistance among and in Ta_3_N_5_ particles. The time constant (*τ*′ = *R*_bulk_*C*_bulk_) of electronic decay in the simulated circuit corresponds to the lifetime (*τ*) determined by TPV. As the bulk capacitance (*C*_bulk_) of the two devices (raw-Ta_3_N_5_ and necked-Ta_3_N_5_) are estimated to be of the same order of magnitude based on eqn (S1) (ESI†) the change in time constant is reflected in the change of resistance in the device. Comparing the lifetime of *τ*_2_ in the two types of Ta_3_N_5_ electrodes as tabulated in Fig. 5b, the smaller *τ*_2_ of the necked-Ta_3_N_5_ electrode means that necking treatment decreases the charge transfer resistance (*R*_bulk_) in the Ta_3_N_5_ film.

The amorphous layer of nitrogen doped tantalum oxide that bridges the Ta_3_N_5_ particles is a plausible route for electron transportation. As necking treatment is performed at high temperature, efficient connection at interfaces of PP will be formed. Additionally, nitrogen doping in the tantalum oxide layer can improve its conductivity^48^ and facilitate the transportation of the photogenerated charges. Indeed, it is reasonable that low charge transfer resistance should lead to the high photovoltage and fast decay process since the Dember photovoltage is caused by drifting apart of electron–hole pairs, the better the conductivity is, the easier they can be dissociated/recombined, and *vice versa*.

**Fig. 6:**
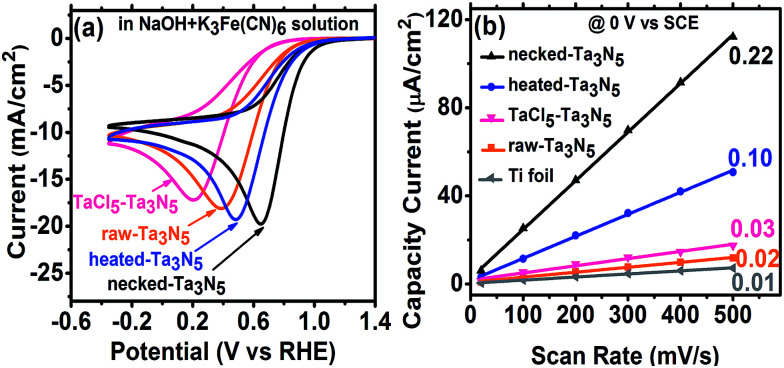
(a) Cyclic voltammograms on raw-Ta_3_N_5_ (orange), TaCl_5_–Ta_3_N_5_ (pink), heated-Ta_3_N_5_ (blue) and necked-Ta_3_N_5_ (black) electrodes for the reduction of ferricyanide in NaOH + K_3_Fe(CN)_6_ aqueous solution. (b) The plots of capacitive current to scan rate for the Ti foil (grey), raw-Ta_3_N_5_ (orange), TaCl_5_–Ta_3_N_5_ (pink), heated-Ta_3_N_5_ (blue) and necked-Ta_3_N_5_ (black) electrodes. The slopes are shown beside the curves.

The efficiency of electron transportation across the interfaces of PP and PS was further probed using the benchmark redox couple of ferri/ferrocyanide.^23^ The exchange of electrons between the solution and electrodes is fast (Fig. S8(a)†), allowing for the characterization of electron transfer by CV. The reduction reaction occurring on the Ta_3_N_5_ electrode (without illumination) can provide a direct measure on the electron transportation. In darkness, the electrochemical reduction occurs by transferring electrons from the Ta_3_N_5_ electrode to K_3_Fe(CN)_6_. Better electron transportation in the film will lead to more facial electron exchange. Moreover, the improved electron transfer in the film will decrease the ohmic polarization of the electrode and decrease the overpotential for the reduction of K_3_Fe(CN)_6_. The CV reveals that Ta_3_N_5_ is poor for hydrogen evolution (Fig. S8(b)†) but active for K_3_Fe(CN)_6_ reduction (Fig. S8(c)†). This allows us to focus on the electron exchange between the electrode and K_3_Fe(CN)_6_ regardless of the influence of hydrogen evolution in the potential window of −0.4 to 1.2 V *vs.* RHE. Fig. S8(c),† shows that the reduction peak of K_3_Fe(CN)_6_ shifts positively on Ta_3_N_5_ electrode, indicating that Ta_3_N_5_ is superior to Ti substrate in electrocatalytic reduction of K_3_Fe(CN)_6_. When Ta_3_N_5_ film has a good contact with the substrate, more electrons will be exchanged with K_3_Fe(CN)_6_ on the Ta_3_N_5_ particles than on the Ti substrate (Fig. S9†). Thus, the results in Fig. 6a can be related to electron transfer in the Ta_3_N_5_ film. It is found that the necked-Ta_3_N_5_ electrode has a reduction current of −19.7 mA cm^−2^ at 0.65 V *vs.* RHE, while that for the raw-Ta_3_N_5_ electrode is −18.1 mA cm^−2^ at 0.38 V *vs.* RHE, which is similar to that of the substrate (Fig. S8(c)†). The more positive potential along with higher current of the reduction peak indicates more efficient electron exchange on necked-Ta_3_N_5_ electrode. Because electrons should transport through the particulate Ta_3_N_5_ film, it is concluded that necked-Ta_3_N_5_ electrode is superior to raw-Ta_3_N_5_ electrode in electron transportation (Fig. S9†). The reduction peak potential of raw-Ta_3_N_5_ electrode (0.38 V *vs.* RHE) is close to the substrate (0.37 V *vs.* RHE), indicating that more electrons are leaked out through the substrate other than through Ta_3_N_5_ because of the weak connection at the PS interfaces. Additionally, the CV of CoPi/necked-Ta_3_N_5_ electrode is similar to that of necked-Ta_3_N_5_ electrode (Fig. S8(d)†), confirming that CoPi has little impact on the charge transfer in the electrode.

To sum up, necking treatment can facilitate charge transfer at the PP and PS interfaces in the Ta_3_N_5_ film which is beneficial for the collection of photogenerated electrons, and depressing the recombination at the interfaces.

For particulate electrode, only the particles having good connection with the conductive layer can provide the area for electrochemical reaction. If an electrode made from powder has good conductivity in the film a porous structure with large electrochemical active area is expected. By eliminating the influence of the diffusion layer with concentrated electrolyte, we can get the relative area of the electrode from its charging capacitance of the Helmholtz double layer based on the following expression:^8,24^

4 *j* = *vC* = (*εS*/4π*d*)*v*

where *v* is the scan rate, and *C* is the capacitance of the double layer which is proportional to the surface area *S*.

Here, we measured the charging current (*j*) in a potential window (−0.1 to 0.2 V *vs.* SCE) where there is no faradaic current at different scan rates without illumination (Fig. S10†). The *j*–*v* plot of each electrode is shown in Fig. 6b and the slope should be proportional to the surface area based on eqn (4). Taking the area of bare Ti substrate as unit, it is found the area only increases a little with TaCl_5_ treatment or heating. However, the refined necking treatment leads to 22 times larger electrochemical surface area. Based on the analysis above, it is inferred that for raw-Ta_3_N_5_ electrode, the poor connection in the particulate Ta_3_N_5_ layer contributes little to the electrochemical active area. Only the layer of Ta_3_N_5_ contact with the substrate can be used for reaction (Fig. S9,† left) and thus the electrochemical area is limited. After necking treatment, the connection at the interfaces of particle–particle (PP) and particle–substrate (PS) are improved substantially and even the layer of Ta_3_N_5_ far from the substrate can be used for reaction (Fig. S9,† right). Thus the necked-Ta_3_N_5_ electrode provides a much larger electrochemical surface area for reaction.

Inspired by the results above, another example was further raised to support the conclusion. We found that the necking treatment can improve the photocurrent to 6.0 mA cm^−2^ at 1.6 V *vs.* RHE for the Ta_3_N_5_ electrodes made from traditional thermal oxidation–nitridation method (Fig. S11, see ESI for details†).

**Fig. 7:**
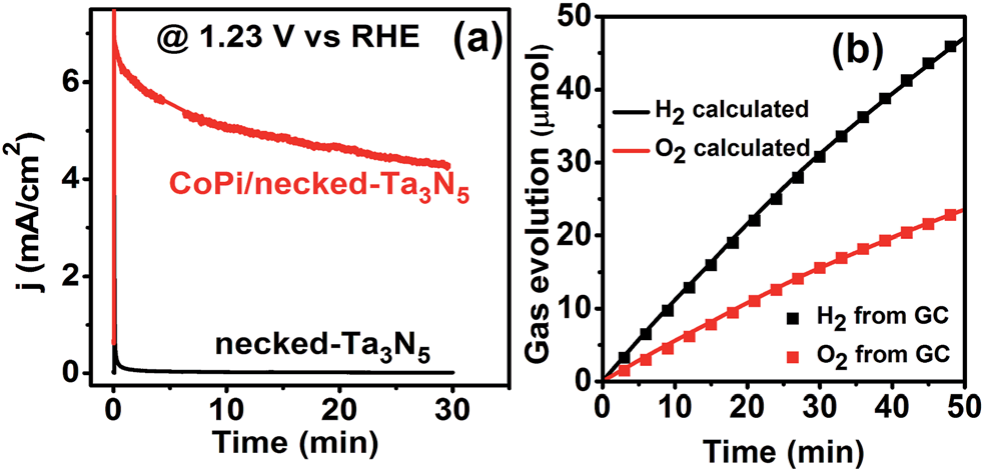
(a) Stability of the necked-Ta_3_N_5_ (black) and CoPi/necked-Ta_3_N_5_ (red) photoanodes at 1.23 V *vs.* RHE. (b) Faradaic efficiency of the CoPi/necked-Ta_3_N_5_ photoanode at 1.23 V *vs.* RHE. Electrolyte: 1 M NaOH aqueous solution. Illumination: 100 mW cm^−2^, Xe lamp (wavelength > 420 nm), area: 0.6 cm^2^.

As for the role of CoPi, it can improve the charge separation by enhancing the charge generation in the Ta_3_N_5_ crystal as shown above. Further, the surface charge injection process is also greatly accelerated with CoPi (Fig. S12†). Because of the cocatalyst promoted charge separation and injection, the CoPi/necked-Ta_3_N_5_ electrode can provide a photocurrent of 11.2 mA cm^−2^ at 1.6 V *vs.* RHE. Moreover, the fast consuming of photogenerated holes can protect Ta_3_N_5_ from being oxidized.^44^ Thus, the stability of the necked-Ta_3_N_5_ electrode is substantially improved with the assistance of CoPi as shown in Fig. 7a. The near unit faradaic efficiency of the CoPi/necked-Ta_3_N_5_ electrode (Fig. 7b) confirms that the photocurrent is originated from O_2_ evolution reaction. However, the photocurrent decayed noticeably with prolonged time (Fig. 7a). The failure of the Ta_3_N_5_ electrode is suspected to be the result of the large surface area of the particulate electrode which makes it difficult to wholly cover Ta_3_N_5_ with effective co-catalyst in the porous structure. Many unconsumed photogenerated holes were accumulated and destroyed the intrinsic Ta_3_N_5_. Further efforts to improve the stability is still in progress.

## Conclusions

Efficient Ta_3_N_5_ photoanode is fabricated on Ti foil by EPD method with refined necking treatment. Further loading cocatalyst CoPi gives a photocurrent of 6.1 mA cm^−2^ at 1.23 V *vs.* RHE under simulated sunlight (AM 1.5 G). To the best of our knowledge, this is the highest photoresponse for an electrode made by EPD. The benefit of necking treatment is proved to stem from the high-temperature treatment and the formation of a nitrogen-doped tantalum oxide layer, which can improve the charge separation efficiency. The TPV shows higher Dember photovoltage and faster decay process for necked-Ta_3_N_5_ electrode, which suggests that necking treatment can decrease the charge transfer resistance at particle–particle interfaces. This will promote the collection of photogenerated charges, decrease the recombination at the interfaces and improve the charge separation efficiency. CV measurement further confirms the benefit of necking treatment in promoting electron transfer and providing higher electrochemically active area for surface water oxidation.

## Supplementary Material

SC-007-C6SC00245E-s001
