# Supplementary material for: Insight into the charge transfer in particulate Ta3N5 photoanode with high photoelectrochemical performance
Source: Chem Sci. 2016 Mar 16;7(7):4391–9. doi: 10.1039/c6sc00245e (PMC6014074; doi:10.1039/c6sc00245e)
Supplement: SC-007-C6SC00245E-s001 [file SC-007-C6SC00245E-s001.pdf]

Electronic Supplementary Material (ESI) for Chemical Science.
This journal is © The Royal Society of Chemistry 2016

Electronic Supplementary Information

Insight into the Charge Transfer in Particulate Ta_3N_5 Photoanode with High Photoelectrochemical Performance

Zhiliang Wang,‡[ab] Yu Qi,‡[ab] Chunmei Ding,[a] Dayong Fan,[ab] Guiji Liu,[ab] Yongle Zhao[ab] and Can Li[a*]

[a]State Key Laboratory of Catalysis, Dalian Institute of Chemical Physics, Chinese Academy of Sciences, Dalian National Laboratory for Clean Energy, The Collaborative Innovation Center of Chemistry for Energy Materials (iChEM), Zhongshan Road 457, Dalian, 116023, China. E-mail: canli@dicp.ac.cn

[b]University of Chinese Academy of Sciences, China.

Preparation of different kinds of metal based substrates

The Pt and Ta based substrates were prepared by sputtering coating. Generally, metal layer with thickness of 3~5 µm was deposited onto a Ti foil (1 cm×2 cm) with the target (Pt or Ta) on a home-built sputtering coater.

Characterization of Photocatalytic water oxidation

The powder (13 mg) of necked-Ta_3N_5 was peeled off from a large piece of electrode (2.7 cm ×2.8 cm). For comparison, similar amount of raw Ta_3N_5 powder (16 mg) was used for the PC reaction.

The photocatalytic reactions were carried out in a Pyrex top-irradiation-type reaction vessel connected to a closed gas circulation system.[1] 100 mL aqueous solution containing a certain amount of photocatalyst was adopted, and 100 mg $AgNO_3$ (Alfa Aesar, ≥ 99.9 %) was used as sacrificial electron acceptor. 0.10 g La_2O_3 (Sinopharm Chemical Reagent, ≥ 99.95 %) was added to keep the pH value of the reaction solution. Prior to irradiation, the reaction mixture was evacuated to make sure that the air was completely removed. Then a 300 W xenon lamp equipped with an optical filter (Hoya, L-42; λ≥ 420 nm) to cut off the ultraviolet light irradiated the system from the top side. A flow of cooling water was used to maintain the system at room temperature. The evolved gases were analyzed by gas chromatography (Agilent; GC-7890A, MS-5A column, TCD, Ar carrier).

Fabrication of Ta_3N_5 device for TPV test

Typically, the Ta_3N_5 device was fabricated by covering the electrode with a piece of FTO (Be careful in case of short circuit). During the experiment, it is found the Ti foil is not flat enough, so we used Ti coated glass as substrate. About 10 µm Ti was sputtered onto the glass (2 cm ×1 cm) in a home-built sputtering coater at the power of 50 W. Then the Ta_3N_5 powder was deposited onto it by EPD. The device was fabricated by clipping a piece of FTO (2.5 cm ×0.7 cm) on the Ta_3N_5 membrane.

And for the device illuminated from frontward and backward direction (Figure S7 a), it is fabricated by depositing raw-Ta_3N_5 on FTO and further covering another pieces of FTO on the electrode.

TPV test is conducted with 355 nm pulsed laser (5 ns). The power is 122 mW unless otherwise mentioned. The signals are connected by an oscilloscope (Tektronix TDS 3012C).

Necking treatment of Ta_3N_5 electrode prepared by oxidation-nitridation

Ta_3N_5 electrode was prepared according to the literature. Typically, Ta foil (2 cm×1 cm) was oxidized at 580 ^oC for 7 min to form a layer of tantalum oxide. The raw Ta_3N_5 electrode was prepared by nitriding of the oxidized Ta foil in NH_3 flow (250 sccm) at 950 ^oC (2 ^oC/min) for 5 h. After that, 20 mM $TaCl_5$ methanol solution was used for necking treatment with 10 µL per time (five times in total). Then the electrode was heated at 600 ^oC (5 ^oC/min) for 1 h in NH_3 flow (100 sccm).

Estimation of C_{bulk} and $C_{crystal}$

C_{bulk} here is defined as capacitive charging to the porous Ta_3N_5 matrix. It can be treated as a parallel capacitor, and the capacitance is determined as:

$$C_{bulk} = \frac{\varepsilon\varepsilon_o}{d} \quad \text{(S1)}$$

The ε is the permittivity constant of Ta_3N_5, estimated to be 17 [2]; ε_o is the vacuum permittivity constant; d is the distance of two substrates, estimated to be 5 µm from SEM image below. And C_{bulk} is estimated to be 3×10^{-5} F/cm^2. C_{bulk} is mainly determined by the intrinsic property of Ta_3N_5 (ε of Ta_3N_5). Thus, we assume that the values of C_{bulk} are at the same order of magnitude for raw and necked Ta_3N_5 devices.

$C_{crystal}$ can be estimated from the τ_1 (τ=RC). Ta_3N_5 has the electric conductivity of 1.3×10^{-4} $(\Omega\ cm)^{-1}$ [3]. Thus, the $C_{crystal}$ is estimated to be at the order of 2×10^{-8} F/cm^2.

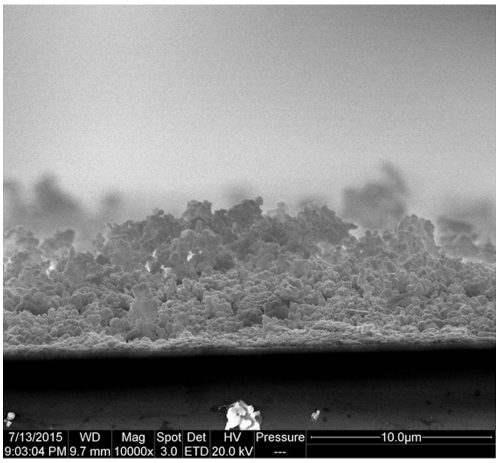

Figure S1. The SEM image of cross-section of necked-Ta_3N_5 electrode.

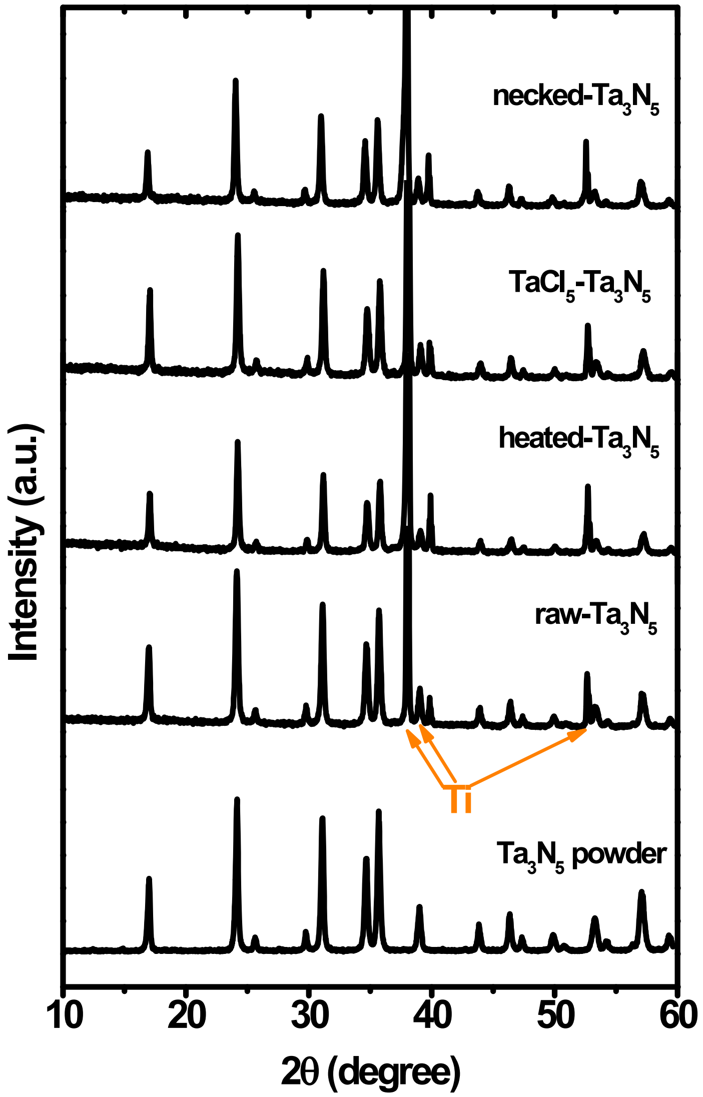

Figure S2. XRD patterns of as-prepared Ta_3N_5 powder and the fabricated electrodes

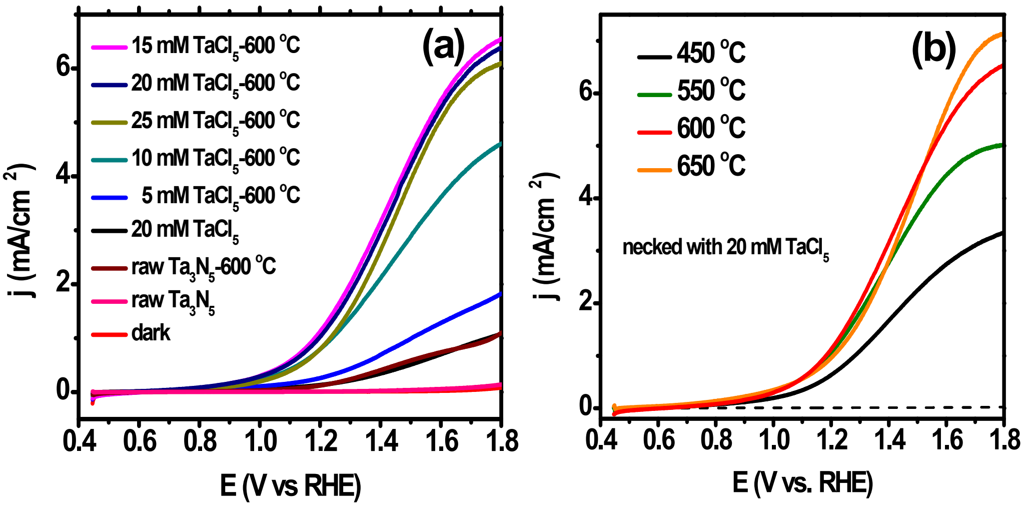

Figure S3. The influences of (a) $TaCl_5$ concentration and (b) necking treatment temperature on the PEC performance of Ta_3N_5 electrodes.

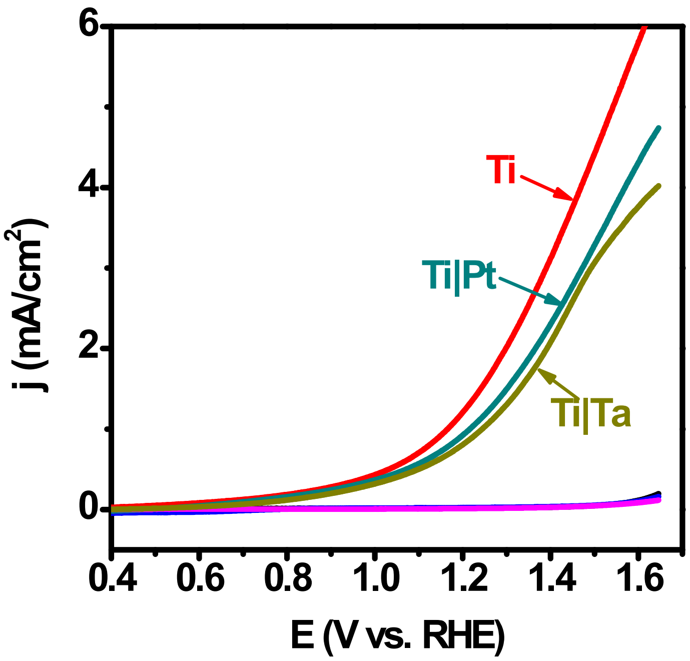

Figure S4. The influence of substrates on the activity. All the Ta_3N_5 electrodes were necked with 20 mM $TaCl_5$ and heated at 600 °C in 100 sccm NH_3 flow.

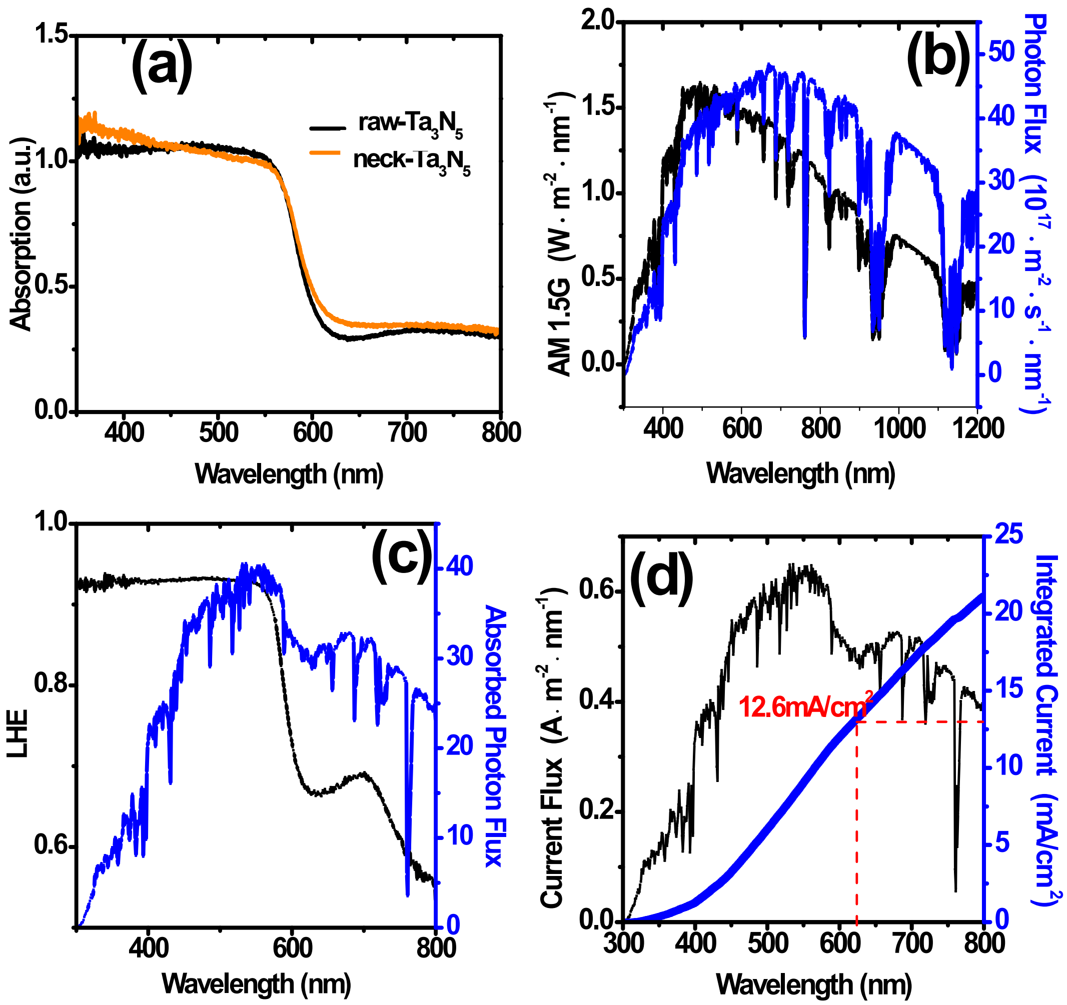

Figure S5. The calculation (blue) of integrated current of Ta_3N_5 electrode. (a) The light absorption spectra of raw-Ta_3N_5 (black) and necked-Ta_3N_5 (orange) electrode. (b) The spectrum of simulated sunlight (AM 1.5 G). (c) The light harvest efficiency (LHE) and absorbed photons. LHE can be got from the absorption with this expression[4]: LHE $(\lambda)=1-10^{-A(\lambda)}$. (d) The calculated integrated current. All the dates are for raw-Ta_3N_5 except that in (a).

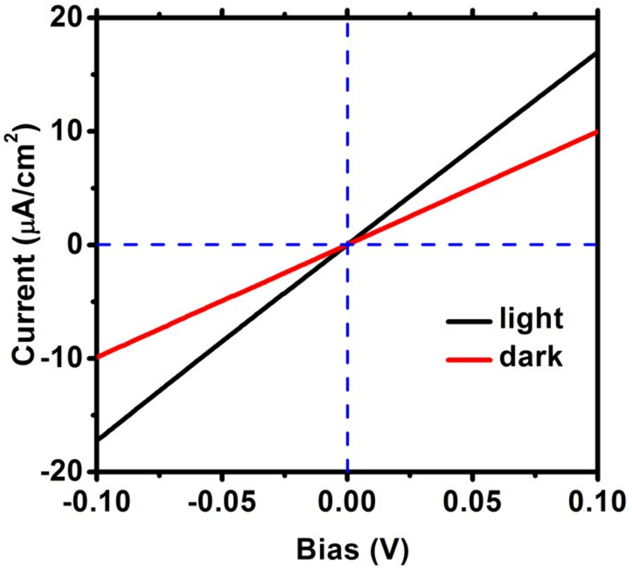

Figure S6. Current-bias response of the Ta_3N_5 device under dark (red) and illumination (black). The light source used here is simulated AM 1.5G sunlight (1 sun).

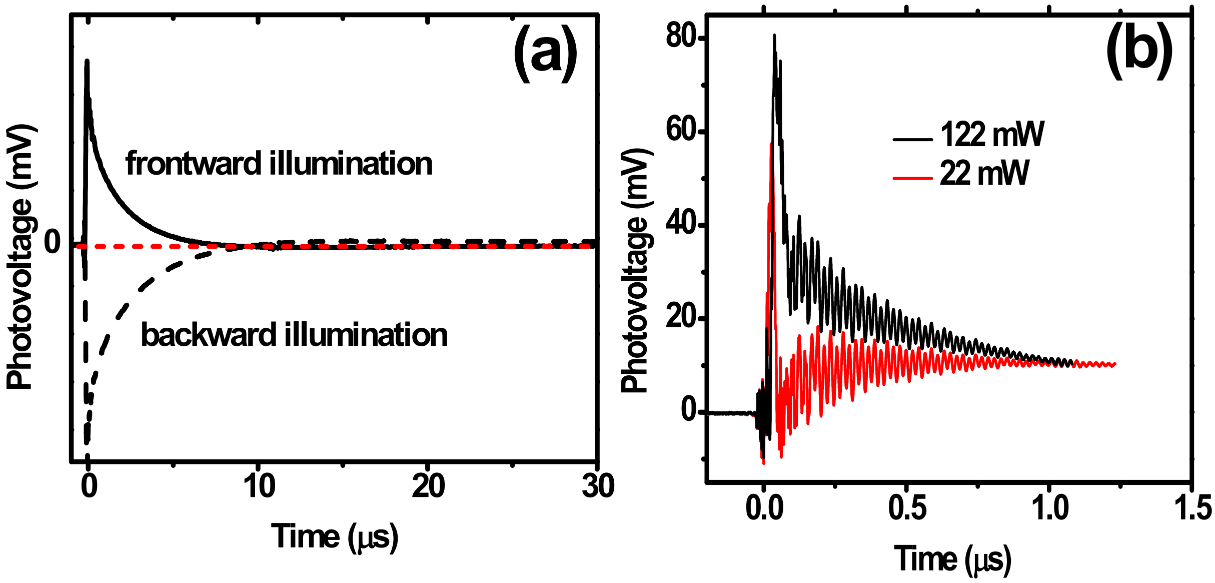

Figure S7. (a) The responses of photovoltage upon frontward illumination (black, solid) and backward illumination (black, dashed line). The red dashed line indicates the 0 mV level. (b) The responses of the photovoltage with different laser power: 122 mW (black) and 22 mW (red)

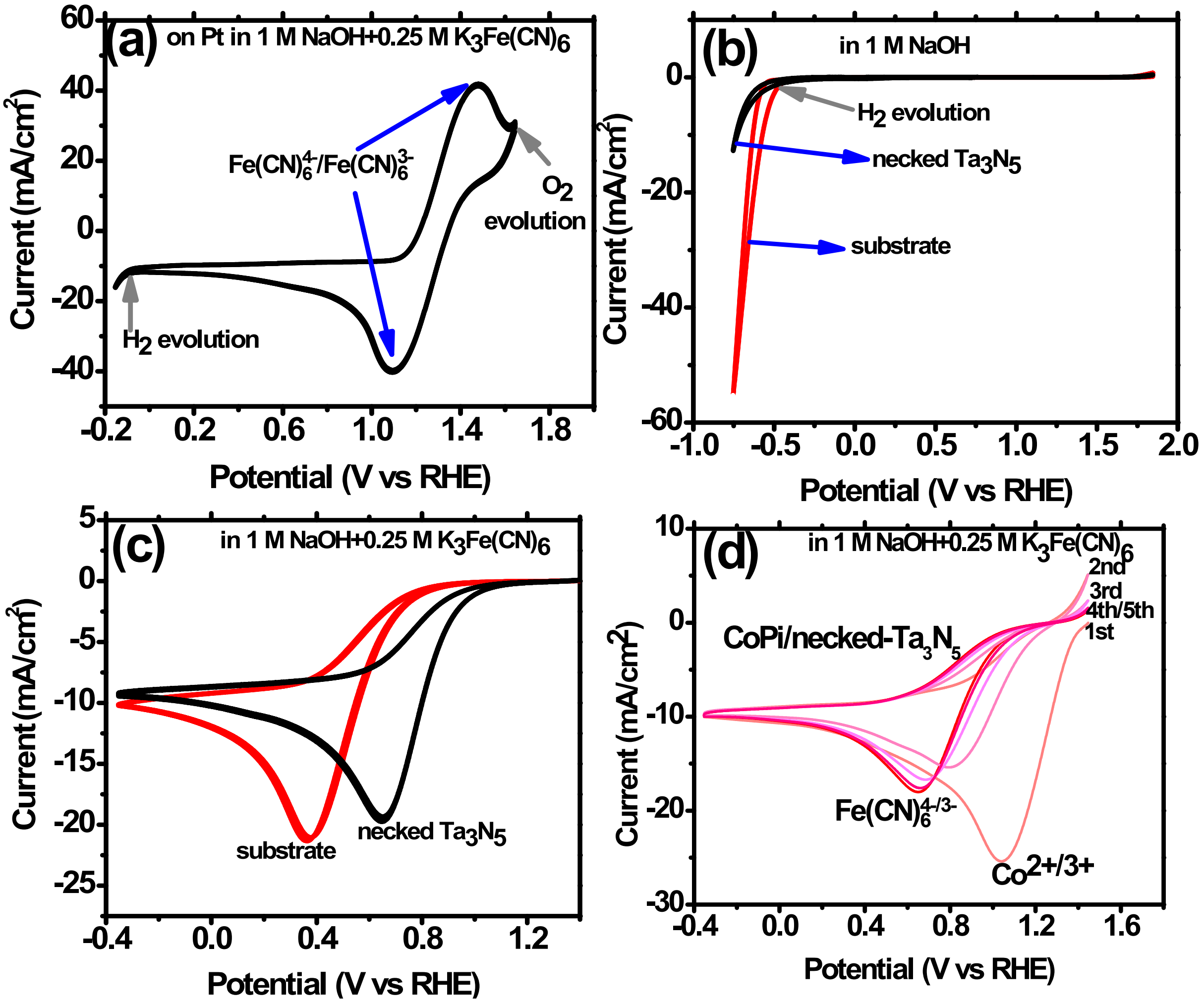

Figure S8. (a) The CV on Pt foil in NaOH (1 M)+$K_3Fe(CN)_6$ (0.25 M) aqueous solution to show the redox peaks of ferri/ferrocyanide. (b) and (c) The CV of necked Ta_3N_5 electrode (black) and its substrate (nitridized Ti foil, red) in NaOH (1 M) and NaOH (1 M)+$K_3Fe(CN)_6$ (0.25 M) aqueous solution, respectively. (d) The CV of CoPi/necked-Ta_3N_5 electrode in NaOH (1 M)+$K_3Fe(CN)_6$ (0.25 M) aqueous solution. The scan is repeated for 5 circles and the first 2 circles is found to be influenced by the reduction of CoPi. At the last circle of scan, the CV curve is similar to that of necked-Ta_3N_5 electrode in the current and potential of the reduction peak.

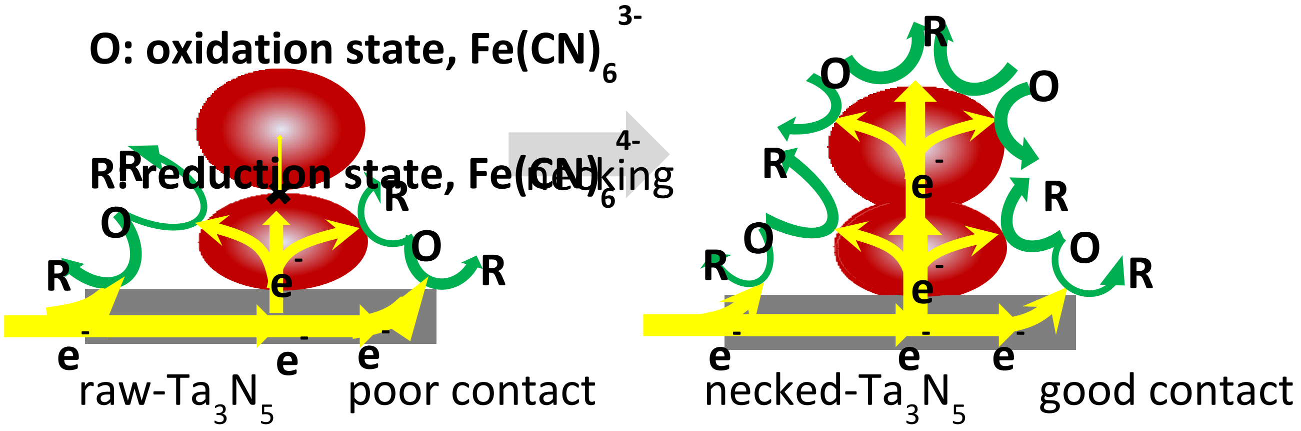

Figure S9. The schematic illustration of electrochemical reduction of $K_3Fe(CN)_6$ on raw-Ta_3N_5 and necked-Ta_3N_5 electrode.

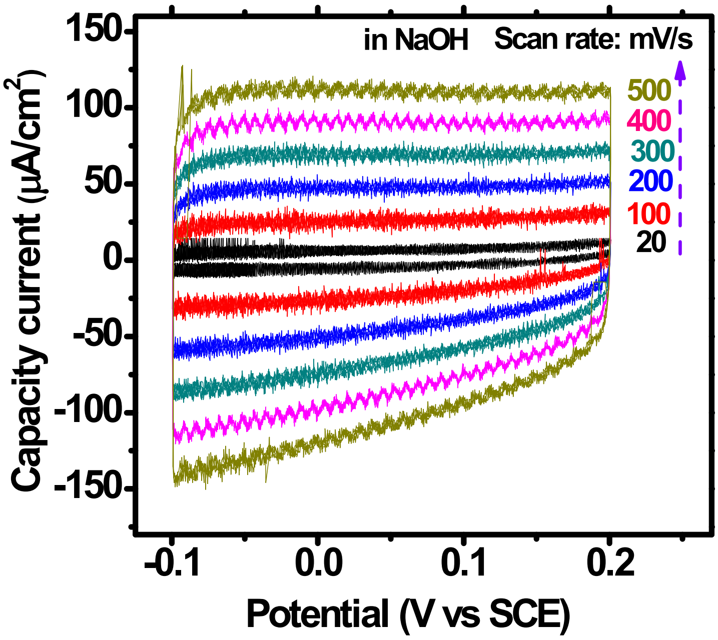

Figure S10. The CV of necked-Ta_3N_5 electrode in NaOH (1 M) at different scan rates (20-500 mV/s) in NaOH (1 M) aqueous solution.

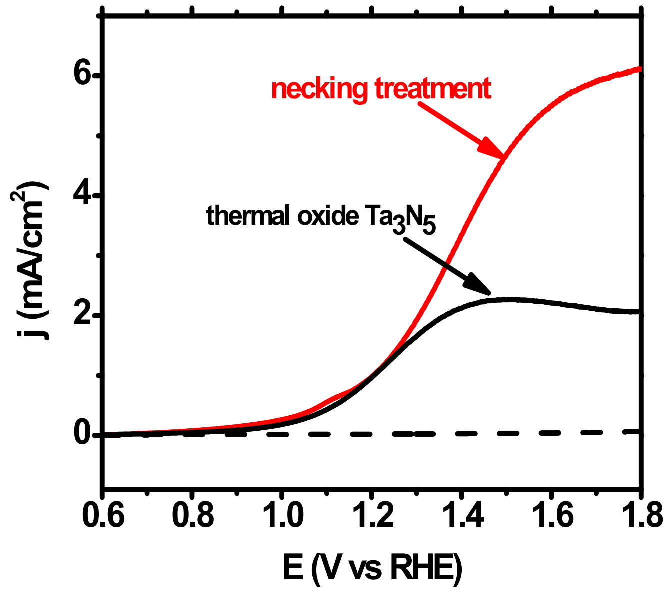

Figure S11. The j-E curves for Ta_3N_5 electrode made from oxidation-nitridation (black) with further necking treatment (red). Electrolyte: 1 M NaOH aqueous solution. Illumination: 100 mW/cm^2, AM 1.5G.

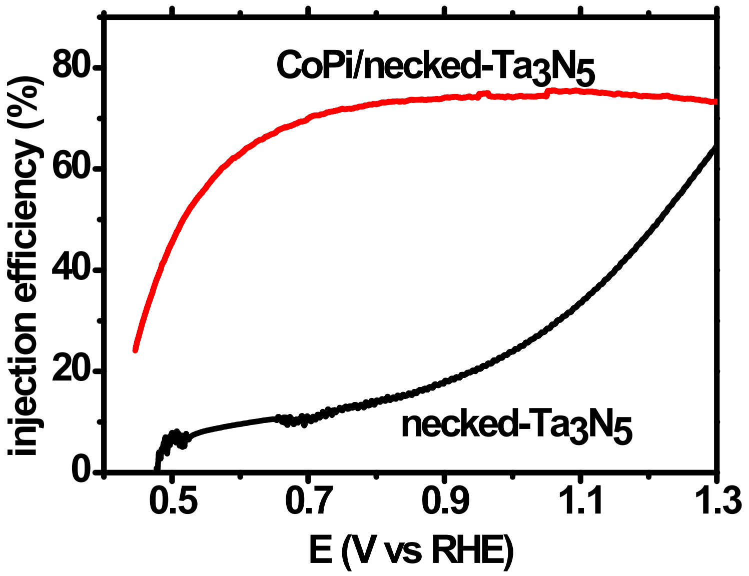

Figrue S12. The injection efficiency of necked-Ta_3N_5 (black) and CoPi/necked-Ta_3N_5 (red) electrodes.

References

1. S. Chen, S. Shen, G. Liu, Y. Qi, F. Zhang and C. Li, *Angewandte Chemie International Edition*, 2015, **54**, 3047-3051.
2. A. Ziani, E. Nurlaela, D. S. Dhawale, D. A. Silva, E. Alarousu, O. F. Mohammed and K. Takanabe, *Physical Chemistry Chemical Physics*, 2015, **17**, 2670-2677.
3. J. Swisher and M. Read, *Metallurgical Transactions*, 1972, **3**, 493-498.
4. T. W. Kim and K.-S. Choi, *Science*, 2014, **343**, 990-994.
